# Evaluating the quality of online resources for patient education on robotic esophagectomy

**DOI:** 10.1007/s11701-025-02297-2

**Published:** 2025-05-22

**Authors:** Susheian Kelly, Rajika Jindani, Sophia Yanis, Noor Habboosh, Priyanka Parmar, Jorge-Humberto Rodriguez Quintero, Tamar Nobel, Marc Vimolratana, Neel Chudgar, Brendon Stiles

**Affiliations:** 1 Department of Cardiothoracic Surgery, Montefiore Medical Center/Albert Einstein College of Medicine, 3400 Bainbridge Avenue, Bronx, NY 10467 USA; 2 Department of Surgery, Montefiore Medical Center, Albert Einstein College of Medicine, Bronx, NY USA

**Keywords:** Robotic, Esophagectomy, YouTube, Educational resources, Patient education

## Abstract

Robotic approaches have gained popularity in recent years, with multiple studies showing improved short- and long-term outcomes with this technique for esophagectomy. Educational resources should be assessed to ensure patients are knowledgeable about the treatment modalities that are available. Our aim is to evaluate whether online content is a reliable source of patient educational material for robotic esophagectomy. A YouTube query was performed for: “Robot Assisted Minimally Invasive Esophagectomy.” The first 60 videos were evaluated by two independent reviewers and scored using the DISCERN tool. Of the 60 videos reviewed, 48 (80%) were included. The average DISCERN score for the videos was 1.3 ± 0.57 (SD), with a score > 3 being good for patient education and ≤ 3 being poor. The content available on YouTube for education about robotic esophagectomy is better suited for surgical education. This underscores a significant opportunity to improve patient education resources for the betterment of shared decision making.

## Introduction

Open esophagectomy is a notoriously invasive procedure with significant post-operative mortality and morbidity, sparking demand for minimally invasive techniques [1]. Robotic-assisted minimally invasive esophagectomy (RAMIE) was first used in 2004 and since then has been used with increasing frequency in candidates for open esophagectomy. RAMIE, like other minimally invasive esophagectomy techniques, confers decreased risk for perioperative complications and a quicker recovery than open esophagectomy [2]. As rates of esophageal carcinoma and pre-cancerous conditions such as Barrett’s esophagus remain high in the western world, the use of minimally invasive esophagectomy such as RAMIE is rising to meet the prevalence of these conditions [3]. It is imperative that candidates for RAMIE are adequately informed about the procedure not only for decision-making purposes, but also for patient empowerment, which has been shown to improve perioperative outcomes [4].

For patients to participate in shared decision-making with their physicians, it is necessary that they have access to adequate education materials on the treatments and procedures they have been prescribed [4]. Surgical patients in particular report feeling dissatisfied with the quality of information they receive pre- and post-op [5, 6]. Accordingly, patients have had to look elsewhere for supplemental medical information. In 2022, the CDC conducted a survey demonstrating that 58.5% of US adults reported using the internet for medical information and additional studies demonstrated the varying quality of the information patients encounter on the internet [7, 8]. The popular video streaming platform,YouTube, has predominated as the preferred self-education tool for patients preparing for surgery [9]. A study using the DISCERN tool to evaluate thoracic surgery videos on YouTube, deemed the repository of relevant videos “poor” for the purpose of patient education [10]. There is a need to investigate the utility of this tool and the quality of educational videos on YouTube for the RAMIE procedure specifically.

Given the importance of patient education about medical procedures and the widespread use of YouTube for patient self-education, it is important to understand how effective existing RAMIE content is at educating patients. In this study, we queried YouTube to compile a database of the most relevant educational videos for the RAMIE procedure. Each video was assessed using DISCERN and the scores were analyzed in aggregate to understand the quality of existing video content. The aim of this study is to bring attention to a possible gap in patient-centered educational RAMIE videos and highlight the potential benefit of bettering available educational RAMIE material on popular platforms like YouTube.

## Methods

### Study design

We designed a cross-sectional, observational study to assess the quality and appropriateness of YouTube videos in educating patients on RAMIE. In September 2023, we queried YouTube for the words “Robotic Assisted Minimally Invasive Esophagectomy” under an “incognito” webpage to minimize the influence of the YouTube recommender system on the video results. The YouTube recommender algorithm gives users video suggestions based on their searches and personal viewing history and is estimated to be responsible for 30% of the videos users view [11]. Minimizing the limiting effects of this algorithm expanded the type and quantity of videos that resulted from the search. We selected the first 60 videos that appeared, given YouTube’s infinite scroll structure, to capture the widest range of videos most likely encountered by patients. We filtered the videos based on relevance to the search term, video format and English language. We excluded videos that were unrelated to robotic esophagectomy and filmed in non-English language. After excluding 12 videos, the remaining 48 videos were included in the analysis.

### Data collection

We recorded descriptive characteristics of the videos such as duration, content, number of views, number of likes, number of dislikes, type of user account and type of video. User accounts were categorized as Publication/educational, Academic institution/hospital, and Individual surgeon. Those that were “Publication/educational” were videos created by journals or other academic groups not affiliated with a university or hospital. Those from academic institutions or hospitals were listed under the institution rather than the individual creating the video. Finally, individual surgeons posted under their own name without any other affiliation.

Two independent reviewers (RJ, NH) evaluated the patient education quality of each video and scored the videos using DISCERN (Table 1). DISCERN is a validated 16-question tool for healthcare workers to evaluate the quality of consumer health information [8–12]. Each of the 16 questions were divided into categories assessing: the reliability of the information, meaning whether it can be trusted as a source for information about treatment options, quality of information, including details on the current treatment option, alternatives, risks and impact on quality of life, and an overall quality rating that summarized the strength of the information source in patient education. In terms of the rating scale, each of the 16 questions were rated on a 5- point scale from 1-No, the criterion was not met, and 5-Yes, the criterion was completely fulfilled. A score < 2 on each question, for example, indicated serious shortcomings, ≥ 3 indicated the material met the requirement for patient education and 5 indicated excellent quality with minimal shortcomings [12].The final DISCERN score was based on a 80-point scale where each of the 16 questions contributed 5 possible total points.

**Table 1:** Quality of Patient Education Material and Corresponding DISCERN Score

Discern Score	Out of 100	Quality Level
64–80	80% & above	Excellent
52–63	65% to 79%	Good
41–51	51% to 64%	Fair
30–40	37% to 50%	Poor
16–29	20% to 36%	Very Poor

To address discordance and reliability in scoring, reviewers practiced scoring a subgroup of videos using the DISCERN handbook guidelines. A wide range of videos representing the different DISCERN scores were evaluated and all questions on how to appropriately apply the DISCERN tool were answered in a focus group setting. Any discordance in scoring was discussed to ensure consistency and accuracy of scores during practice sessions and during the formal video assessment.

### Data analysis

Statistical analysis was performed using IBM SPSS Statistics for Macintosh, version, 29.0.0.0 (IBM Corp.). Descriptive statistics was used to characterize the videos in terms of mean DISCERN score, standard deviation and percentages of each video type. Significance was set at *P* < 0.05.

## Results

### Video demographics

Of the 60 videos reviewed, 48 (80%) were included in the study. The videos had a mean duration of 13.3 min (SD = 18.1), with 4,217.4 average views (SD = 17,676.1) and 21.4 average likes (SD = 80.9). Most videos were published by accounts in the USA (56%), then Asia (22.9%) and Europe (12.5%). The most common account type were individual surgeons who published 18/48 videos (38%) with 1,001 views on average and 8 average likes (Table 2). Publication/educational groups were the second most frequently published video type with 16/48 videos (33%), 2,302 average likes and 11 average views. Academic programs/hospital groups published the least number of videos,14/48 videos (29%), but had the highest average views of 10,338 and highest average likes of 48.

**Table 2:** Differences in Viewer Metrics and DISCERN Score by Type of Publisher Account on YouTube

Type of Account	Total # of videos	Average views	Average likes	Average dislikes	DISCERN Score Q16: Overall quality rating (1–5)
Publication/Education group	16 (33%)	2302	11	0	1.2
Academic/hospital	14 (29%)	10,338	48	0	1.8
Individual Surgeon	18 (38%)	1001	8	0	1.1

In terms of the content of the videos, 28/48 (58%) were purely intraoperative demonstrations and 4/48 (8%) included case presentations. 7/48 (15%) of the videos alluded to at least one component of the treatment pathway/algorithm, but none offered a comprehensive explanation of the treatment approach for RAMIE. A minority of videos were patient case presentations from which viewers could gain limited insight into an individual’s experience undergoing RAMIE. However, videos overall did not cover a stepwise shared decision-making approach to undergoing this major surgery.

### Video scoring

Using the DISCERN tool for video scoring, the final question, Q16, summarized the overall quality of the educational material on a scale from 1–5. The average DISCERN score for Q16 was 1.3 ± 0.57 (SD), with a score > 3 being good for patient education and a score ≤ 3 being poor. Only one video scored higher than a 3. The metrics and reliability of videos in terms of their DISCERN score varied by publisher type. Videos published by academic groups/hospitals had the highest average DISCERN score for Q16 of 1.8 while videos by Publication/educational groups had an average score of 1.2. Individual surgeons who published the most videos had the lowest average DISCERN score of 1.1. Overall, the less frequently published videos by academic groups/hospital were associated with the highest DISCERN scores.

## Discussion

Robotic assisted minimally invasive esophagectomy (RAMIE) is being offered to patients as an alternative to the more invasive open esophagectomy. Many studies have found that robotic-assisted surgery has technical advantages especially in narrow operative fields such as the mediastinum and can improve oncological outcomes by facilitating larger lymph node harvest and complete R0 resection [13–15]. Robotic-assisted surgery also has direct patient benefits as randomized controlled trials and meta-analyses have found less blood loss, lower rates of pulmonary and cardiac complications, less pain, improved short term quality of life and better post-operative functional recovery [16, 17]. Due to increased utilization of robotic-assisted surgeries and its frequent inclusion as a treatment option during shared decision-making discussions between patients and their surgeons, high quality educational resources to ensure patients are knowledgeable about this treatment modalities, is paramount.

High quality information is central for patient engagement in their care. With the increased utilization of the internet, mobile devices and social media, patients can now access health information from anywhere to help guide their medical decisions. Receiving information from their clinician is only the first step to becoming informed as many patients use a combination of approaches. Data from the 2022 National Health Interview Survey, found that 58.5% of patients used the internet to look for health and medical information [7]. With the internet, patients have increased risk of being exposed to inaccurate or biased health information. Indicators of quality health information on the internet include accuracy, completeness and currentness of information [18]. High quality educational materials should mirror the principles of informed decision making which include discussion of patients’ role in the decision-making process, the clinical issue and details of the treatment options including alternatives and risk factors, and discussion of areas of uncertainty or evolving research [19, 20].

The collaborative partnership between informed patients and their clinicians promotes patients’ health and wellbeing, facilitate better decision making and sustains ongoing interest in their care [21]. Patients only spend limited time in health care facilities with their treatment providers and frequently are on their own when making medical decisions. Therefore, there is a practical need for accurate, high quality medical information on the internet and on social media.

YouTube is the most widely used video- sharing platform, with over 2.7 billion users worldwide as of 2023. This study evaluated whether this online platform is a reliable source of patient educational material for robotic esophagectomy. Our study found that most of the content available on YouTube on this topic was published by individual surgeons whose videos had the lowest average DISCERN scores, averages like and average views. Similar studies have also found that YouTube has poor quality educational videos with high levels of bias and many of these videos were uploaded by physicians [22]. Low average likes and views lend support to the opinion that physician videos appear to be intended more for surgeons, clinicians and surgeon trainees and are not very impactful in terms of patient engagement. These types of videos include intraoperative demonstrations but generally fail to capture the global treatment pathway for esophageal cancer which would be helpful for patients making treatment decisions. Overall, this type of content produced by surgeons for education about robotic esophagectomy is better suited for surgical education.

Academic groups and hospitals produced the least amount of educational YouTube videos on RAMIE but had more engagement in terms of having the highest number of averages likes and views and the highest average DISCERN score. It could be postulated that these facilities have both the incentive and resources to produce higher quality videos geared towards patient education on key components of the treatment pathway. More informed patients can increase the likelihood of undergoing treatment with better preparation and compliance.

Though hospitals and academic groups appear to produce more impactful educational videos for patients, the overall quality of educational videos for RAMIE on YouTube is low. Only one video scored > 3 on Q16 and the average score overall was 1.3/5. These findings mirror those in a similar study that assessed the quality of educational YouTube videos for the most common thoracic surgery procedures. They found similar inadequacy in the quality of healthcare videos [10]. These findings underscore the need for more high-quality patient educational resources for the betterment of shared decision making on high-risk surgical procedures.

If used effectively by surgeons, clinicians and healthcare facilities, YouTube could be a beneficial tool to continue engaging patients outside of treatment settings. This critical resource could be capitalized upon to provide timely access to educational information, ensuring that the information meets the standards of high-quality educational material and aligns with shared decision-making principles [23]. As information becomes increasingly accessible to patients, involvement of health professionals in the production and dissemination of quality educational videos can help patients navigate the wealth of biased misinformation on social media platforms like YouTube. Clinicians could also build sustainable relationships with community partners, content creators and patients to increase the availability and reach of quality medical information.

### Limitations

This study has several limitations. The sampling method of choosing the first 60 videos that resulted from the YouTube search may not adequately capture all YouTube videos on RAMIE. However, the videos selected likely represent the most frequently watched videos by patients on this topic and selecting 60 videos likely captures more videos than an individual patient may watch for education on the procedure. Our search term, “Robotic Assisted Minimally Invasive Esophagectomy” may differ from other search terms that patients may use, such as “robotic surgery”, robotic esophagectomy” and “minimally invasive esophagectomy.” However, searching the accurate procedure name allows us to select the videos most relevant to our research topic and filter out alternative treatment options such as laparoscopic or open esophagectomy. As different forms of social media become more popular, YouTube may become decreasingly utilized for patient education as patients may switch to newer platforms like Instagram and TikTok. However, for now, YouTube remains highly utilized especially for long-form video content where detailed information is needed on important topics like surgery.

The DISCERN tool was designed to evaluate the quality of written consumer based educational content, not necessarily videos, and its application in this context may not be perfectly fitted to its intended use. However, this is the most well studied and validated tool available to assess the quality of consumer health information. Additionally, by having two trained reviewers who scored the videos using the DISCERN tool, we limited bias and errors in utilization of this instrument.

## Conclusion

This is the first study to assess the quality of social media educational content on RAMIE on the YouTube platform. We found that most videos overall, had low quality of educational content and captured only a limited section of the global treatment algorithm involved in esophageal surgery. This remained true even if videos were produced by surgeons, hospitals and other members of the treatment team. This underscores a significant opportunity for treatment providers and facilities to become intentionally engaged in the production of patient-centered, high quality, health education content that continues the process of shared decision making even outside of the clinical setting.

## Data Availability

No datasets were generated or analysed during the current study.
