# Correction: Evaluating the quality of online resources for patient education on robotic esophagectomy

**DOI:** 10.1007/s11701-025-02452-9

**Published:** 2025-06-25

**Authors:** Susheian Kelly, Rajika Jindani, Sophia Yanis, Noor Habboosh, Priyanka Parmar, Jorge Humberto Rodriguez-Quintero, Tamar Nobel, Marc Vimolratana, Neel Chudgar, Brendon Stiles

**Affiliations:** 1 Department of Cardiothoracic Surgery, Montefiore Medical Center/Albert Einstein College of Medicine, 3400 Bainbridge Avenue, Bronx, NY 10467 USA; 2 Department of Surgery, Montefiore Medical Center, Albert Einstein College of Medicine, Bronx, NY USA

**Correction: Journal of Robotic Surgery (2025) 19:228** 10.1007/s11701-025-02297-2

In the original version of this article, the author’s name Jorge Humberto Rodriguez-Quintero was incorrectly written as Jorge‑Humberto Rodriguez Quintero.

